# Translation, validity, and reliability of an Arabic version of the short Healthy Eating Index tested in young Saudi adults: a questionnaire-based study

**DOI:** 10.3389/fpubh.2025.1581863

**Published:** 2025-05-23

**Authors:** Abeer Salman Alzaben, Nahla Mohammed Bawazeer

**Affiliations:** Department of Health Sciences, College of Health and Rehabilitation Sciences, Princess Nourah Bint Abdulrahman University, Riyadh, Saudi Arabia

**Keywords:** Saudi, adults, nutritionist, reliability, Healthy Eating Index (sHEI), translation, validity

## Abstract

**Introduction:**

The short Healthy Eating Index (sHEI) effectively evaluates overall and specific aspects of dietary quality; however, an Arabic version has not been developed yet. This study aimed to translate the sHEI into Arabic, adapt it culturally, and assess its reliability and validity among young Saudi adults.

**Methods:**

The 22-item sHEI was translated and reviewed by a panel of nine Saudi nutritionists and dietitians for face and content validity. Reliability was assessed using an online, self-administered questionnaire completed by 615 participants recruited through convenience sampling. Internal consistency was evaluated with Cronbach’s alpha, and construct validity was assessed through factor analysis using Bartlett’s test of sphericity and the Kaiser–Meyer–Olkin measure of sampling adequacy.

**Results:**

Face validity analysis indicated a 90% comprehensibility score, whereas content validity values for individual items ranged from 0.89 to 0.99, with a mean expert endorsement between 0.98 and 1.00. The sHEI nutrition scale achieved a Cronbach’s alpha of 0.81 for adequacy-related items after excluding Question 22 (water intake). Principal component analysis identified three factors related to adequacy and one factor related to moderation.

**Discussion:**

The Arabic version of the sHEI demonstrated strong validity and reliability for assessing dietary quality among young Saudi adults. It provides a practical tool for use in community-based assessments, nutritional interventions, and future research.

## 1 Introduction

The relationship between dietary patterns and health outcomes is multifactorial, driven by the synergistic effects of various nutritional components rather than isolated dietary elements. Diets characterized by a high intake of ultra-processed, energy-dense foods with low nutritional value have been strongly implicated in the pathogenesis of non-communicable diseases, including obesity, type 2 diabetes mellitus, and cardiovascular diseases (1). Conversely, adherence to a balanced, nutrient-dense diet is recognized as a pivotal preventive measure against these conditions (1), which continue to show an upward global trajectory in prevalence (2).

Recent trends in nutritional epidemiology have shifted from focusing solely on individual nutrients to evaluating the effects of whole foods and dietary patterns on health outcomes. In this context, dietary quality (DQ) serves as a comprehensive assessment, accounting for both food groups and nutrient intake (3–5). Suboptimal DQ is characterized by insufficient consumption of essential micronutrients and an excessive intake of hypercaloric foods rich in saturated fats, added sugars, and refined carbohydrates. Furthermore, poor DQ typically indicates an imbalance in food variety and non-adherence to dietary guidelines that emphasize the consumption of fruits, vegetables, whole grains, lean proteins, and other nutrient-dense foods essential for optimal physiological function. Consequently, low DQ is associated with an elevated risk of metabolic disorders, obesity, and chronic diseases (6). In contrast, high DQ has been shown to confer a protective effect against cardiometabolic diseases, including type 2 diabetes, by modulating risk factors and promoting metabolic health (6).

Thus far, a validated DQ tool has not been used in studies from Saudi Arabia. Several studies in Saudi Arabia have used nutrient intake and dietary patterns as surrogate markers of overall DQ (7, 8). Therefore, developing a validated DQ tool is important to measure the association between overall DQ and other health factors. Due to the strong association between diet and the risk of obesity and chronic diseases, DQ tools (such as the Healthy Eating Index (HEI) and Diet Quality Index [DQI]) were developed to meet the dietary recommendations, aiming to reduce the risk of chronic diseases (5). For example, DQI was developed in 1994 based on the dietary recommendations of the National Research Council Food and Nutrition Board in 1989 and focused on only 8 components (9). In addition, the HEI is one of the most used DQ tools in different Arab populations (10–12). Several studies have adapted the HEI based on the national nutritional guidelines of the countries (10–12).

Given the need for a more efficient tool to assess DQ without compromising validity, a short version of the HEI (sHEI) has been developed (13). The sHEI includes a subset of the original HEI components and has demonstrated reliability in evaluating overall DQ as well as individual food items (13). Shatwan and Alzharani (14) used the sHEI in college students in Saudi Arabia; however, no studies have validated the sHEI to assess DQ in Saudi Arabian, Arabs, or other populations, particularly among young adults. Moreover, although versions of the HEI have been applied in some Arabic-speaking countries (15, 16), they were often utilized without prior cultural adaptation or formal validation, highlighting the need for a validated tool like the sHEI in these populations.

In Saudi Arabia, the established nutritional guidelines, provided by the Saudi Ministry of Health and the Saudi Food and Drug Authority, align with international standards, emphasizing balanced nutrition and appropriate portion sizes (17). The ‘gold standard’ for validating new dietary assessment tools typically involves comparisons with established methods, such as dietary recalls or biomarker analyses.

Accordingly, this study aimed to translate the sHEI into Arabic, validate its content, and assess its reliability among young adults in Saudi Arabia. Assessing the validity and reliability of the sHEI is crucial to ensuring consistency, improving data quality, and enhancing generalisability by increasing external validity. We hypothesized that the Arabic version of the sHEI would demonstrate adequate reliability and validity for assessing DQ in accordance with the established nutritional guidelines. This tool holds significant potential for use in community-based assessments and nutritional interventions.

## 2 Materials and methods

### 2.1 Participants and sample size

This study employed a convenience sample of male and female university students aged 18 to 25 years residing in Saudi Arabia. Data collection occurred between December 2022 and April 2023. According to the General Authority for Statistics, the young adult population in Saudi Arabia is 4,956,921. Based on sample size calculations using epidemiological equations (18), with a significance level of *p* ≤ 0.05 and assuming a prevalence rate of 50%, a minimum sample size of 385 individuals was required. A non-probability sampling method was used to recruit participants (19). The study was approved by the Institutional Review Board (IRB) of Princess Nourah Bint Abdulrahman University, Riyadh, Saudi Arabia (IRB approval number 22–0935, dated 7 November 2022). Informed consent was obtained electronically, with participants consenting by selecting the ‘I agree’ option in the online questionnaire.

### 2.2 Design of the short healthy eating index

The sHEI consists of 22 items assessing DQ across multiple food groups (20). These items are grouped into 13 components: fruits, vegetables, whole grains, proteins, seafood, plant proteins, refined grains, nuts and seeds, sugar-sweetened beverages, added sugar, sodium, saturated fats, and water. Participants reported their daily intake using seven response options, ranging from ‘less than one’ to ‘six or more’ servings. Frequency-based questions offered five options, including ‘a couple of times per week’, ‘a couple of times per month’, ‘a couple of times per year’, ‘almost never’, and ‘never’. For daily intake levels, responses included ‘none/almost none’, ‘some’, and ‘a lot’.

### 2.3 Translation and cultural adaptation

The original sHEI, validated in English by Colby et al. (13), was translated into Arabic, following established guidelines for conceptual equivalence. Native Arabic-speaking researchers with a health care background performed the initial forward translation approved by the original authors, emphasizing clarity and cultural relevance. The back-translation was conducted by independent bilingual experts to verify consistency with the original version.

A panel of two nutrition experts reviewed the back-translated version, ensuring alignment with the original instrument. Cultural adaptations were made to reflect regional dietary habits. For example, items such as watercress, parsley, and leek were included under ‘green vegetables’, and local types of bread were specified under the ‘grains’ section.

Additionally, ounces were converted to grams, fluid ounces to millilitres, and references to objects like ‘baseballs’ were replaced with familiar measures, such as ‘spoons’ or ‘cups’. The option ‘choose not to answer’ was removed to prevent missing data and ensure completeness of responses.

The final Arabic sHEI underwent pre-testing to enhance semantic and content equivalence. A panel of nine Saudi nutritionists and dietitians from both academic and clinical backgrounds reviewed the instruments. These experts, none of whom were involved in the initial translation process, evaluated each item for clarity, relevance, and articulation on a 5-point Likert scale, following the recommendations of Sousa and Rojjanasrirat (21). Items with average scores below 3 were revised accordingly.

### 2.4 Validity testing

The same expert panel assessed the face and content validity of the Arabic sHEI. Face validity ensured that the instrument was understandable and engaging for the intended respondents, using Yusoff’s evaluation framework (22). These assessments included:

(a) Content completeness: experts evaluated whether the questionnaire covered all key aspects of dietary assessment, indicating missing elements when necessary.
(b) Comprehensibility: the clarity of the questions was assessed, with unclear items flagged for revision.
(c) Time to complete: experts rated the time required to complete the questionnaire on a scale of 0 to 10 (0 = ‘unacceptably long’; 10 = ‘completely acceptable’) (23, 24).

Content validity was established by calculating the Content Validity Index (CVI) (25). Each item was evaluated by assigning scores ranging from 1 (indicating irrelevance) to 5 (indicating high relevance), with scores of 4 or 5 categorized as relevant (coded as ‘1’), and scores of 1 to 3 as irrelevant (coded as ‘0’).

The experts further reviewed the relevance of each question on a 5-point Likert scale, with ‘1’ representing ‘not at all relevant’; ‘2’, not relevant; ‘3’, neutral; ‘4’, relevant; and ‘5’, ‘very relevant’. Questions with scores of 4 and 5 were considered relevant. The Scale Content Validity Index (S-CVI) was calculated using both the average and universal agreement methods outlined by Rodrigues et al. (25).

The Item Content Validity Index (I-CVI) was determined by the proportion of experts who rated an item as ‘relevant’ divided by the total number of experts in the panel. An I-CVI exceeding 0.78 indicates sufficient relevance. When five or fewer experts unanimously agreed on an item’s relevance, the I-CVI was assumed to be 1.0. If six or more experts agreed, with an I-CVI ≥ 0.78, the item was also deemed relevant, following the criteria established by Polit and Beck (26).

Construct validity examines whether a tool accurately measures the concept it is intended to assess. It was assessed statistically using factor analysis, with Bartlett’s test of sphericity applied to assess the correlation matrix of the sHEI components. The Kaiser–Meyer–Olkin (KMO) measure was used to evaluate the sampling adequacy for each component (27, 28).

### 2.5 Reliability testing

Reliability was assessed by re-administering the questionnaire to determine the consistency and stability of participants’ responses based on methods outlined by Tsang et al. (29). Test–retest reliability was evaluated by administering the questionnaire twice, with a two-week interval, among 20 individuals from the representative sample. A convenience sample of 615 male and female participants was used for the reliability analysis.

### 2.6 Statistical analyses

Data were analysed using IBM SPSS (version 26.0, IBM Corp., Chicago, IL, USA). Continuous variables were presented as means ± standard deviations, whereas categorical variables were expressed as frequencies and percentages. Internal consistency was evaluated using Cronbach’s alpha. Construct validity was assessed through factor analysis with Bartlett’s test of sphericity and KMO measure of sampling adequacy. A KMO value between 0.8 and 1.0 was considered satisfactory (27, 28). Principal component analysis with varimax rotation and Kaiser normalization was applied, excluding factor loadings below 0.3, with factors loading above 0.3 considered significant for component interpretation (30). Statistical significance was set at *p* < 0.05.

## 3 Results

### 3.1 Face validity

Table 1 summarizes the findings for face validity. A total of 78% of expert nutritionists, including PhD holders who are faculty members and/or experienced clinical dietitians and nutritionists, considered the questionnaire to be sufficiently complete. While some experts recommended including additional food items, such as junk food, fast food, and fried foods, and substituting ‘small plate’ for ‘cup’ in relevant questions, these changes were not implemented to maintain the accuracy and integrity of the original translation. Comprehensibility was rated favorably, with 90% of experts finding the questionnaire easy to understand. A recurring suggestion was to incorporate culturally specific Saudi foods into the food group categories. Additionally, experts recommended clarifying the terms ‘almost none’, ‘some’, and ‘a lot’ in Questions 20 (added sugar) and 22 (water intake). These terms were refined by including portion sizes based on dietary intake studies conducted among Saudi adults (31). The questionnaire’s completion time received positive feedback, with a mean score of 8.8 ± 0.9 on a 10-point scale, indicating that the time required to complete the survey was acceptable.

**Table 1:** Face validity (*N* = 9 experts).

Item	Results
Completeness of content	78%
Comprehensibility	90%
Time for completion (mean ± SD)	8.8 ± 0.9

The number of experts (*N*) expressing full agreement was used to calculate the mean and SD, using a scoring system from 0 to 10.

### 3.2 Content validity

The I-CVI for individual items ranged between 0.89 and 0.99, confirming that each item met the required threshold for validity. The S-CVI for universal agreement (S-CVI/UA) also demonstrated satisfactory relevance and articulation, although it was slightly lower in terms of precision and understandability. The mean proportion of experts endorsing each item as relevant ranged from 0.98 to 1.00 (Table 2).

**Table 2:** Content validity.

Item	Mean I-CVI	S-CVI/UA	Mean expert proportion
Relevant	0.99	0.91	1.00
Precise	0.89	0.82	0.98
Well-articulated	0.99	0.91	1.00
Understandable	0.98	0.86	1.00

I-CVI, Item Content Validity Index; S-CVI/UA, Scale Content Validity Index for universal agreement.

### 3.3 Reliability

A total of 615 participants were included in the reliability analysis, with a mean age of 23.0 ± 7.6 years. The sample consisted of 11.4% males and 88.6% females, with 77.2% being students. The KMO measure of sampling adequacy was 0.84, indicating adequate sampling, and Bartlett’s test of sphericity was significant (*p* < 0.0001; Table 3). The internal consistency of adequacy-related items on the sHEI was assessed using Cronbach’s alpha, yielding a value of 0.81 after excluding Question 22 (water intake). However, Cronbach’s alpha for moderation-related items was 0.44, indicating lower consistency within this domain.

**Table 3:** Kaiser–Meyer–Olkin measure and Bartlett’s test.

Item		Value
Kaiser–Meyer–Olkin measure of sampling adequacy		0.84
Bartlett’s test of sphericity		
	Approx. chi-square	2,293.564
	Df	105
	Sig.	< 0.0001

Df, degree of freedom; Sig., significance.

### 3.4 Factor loading analysis

Factor loading analysis was conducted on 21 items from the sHEI nutrition scale, identifying four distinct factors. Items with higher absolute factor loadings were considered to have a greater contribution to the respective components. Factor 1 represented items related to seafood, beans, nuts/seeds, low-fat milk, and fruit juices and was inversely related to sugar-sweetened beverages. Factor 2 included loadings from milk, grains, whole grains, low-fat milk, and a variety of vegetables and beans. Factor 3 was primarily associated with all types of fruits and vegetables. Factor 4 comprised moderation-related items, including added sugar, saturated fat, and sugar-sweetened beverages. The cumulative variance explained by these four factors was 56.7%, with individual contributions of 15.5, 15.3, 15.1, and 10.8%, respectively (Table 4).

**Table 4:** Factor loadings across the sHEI nutritional scale.

sHEI item	Factor 1	Factor 2	Factor 3	Factor 4
Q16_seafood	0.741			
Q14_beans	0.672	0.345		
Q15_nuts/seeds	0.659			
Q10_milk		0.731		
Q6_grains		0.675		
Q8_whole grains		0.610		
Q12_low fat milk	0.520	0.546		
Q5_starchy vegetables		0.451	0.446	
Q3_vegetables		0.367	0.740	
Q4_green vegetables		0.305	0.721	
Q1_fruit			0.679	
Q2_fruit juices	0.421		0.586	
Q20_added sugar				0.807
Q21_saturated fat				0.745
Q18_SSB	−0.436			0.543

sHEI, short Healthy Eating Index; SSB, sugar-sweetened beverages.

Extraction method: principal component analysis.

Rotation method: Varimax with Kaiser normalization.

Iterations: 10.

Total cumulative variance: 56.69%.

For simplicity, factors loading below 0.3 are not shown.

## 4 Discussion

This study aimed to translate, validate, and assess the reliability of the Arabic version of the sHEI for use among young Saudi adults. The tool achieved satisfactory reliability and validity scores, confirmed by a panel of nine nutrition and dietetics experts. This process builds on the original sHEI, which was developed and validated in English using 24-h dietary recalls; however, the original tool’s reliability was not extensively evaluated (13).

The HEI, widely recognized as a comprehensive measure of DQ, was initially developed by Kennedy et al. (32) to assess adherence to US dietary guidelines. Over the years, the HEI has undergone several revisions to reflect updates in nutritional recommendations and to address the rising prevalence of chronic diseases (33). Given the need for simplified, reliable tools to assess DQ across diverse populations, the sHEI provides a practical solution by evaluating two critical components of dietary behavior: adequacy (intake of nutrient-rich foods) and moderation (limiting added sugars, saturated fats, and other unhealthy components) (13, 34–37).

The Arabic version of the sHEI retained the original structure of 22 questions, with 18 questions assessing the intake of primary food groups (e.g., vegetables, fruits, grains, dairy, meats, and legumes), 3 items focused on added sugars and saturated fats, and 1 item measuring water intake (13). Cultural adaptations were made to align the tool with Saudi dietary patterns, such as the inclusion of commonly consumed local vegetables and bread types (31). Additionally, portion sizes were clarified for questions on added sugar and water intake to improve accuracy.

The face and content validity assessments revealed that the Arabic sHEI is a comprehensive and well-adapted tool for the Saudi population. The expert panel rated the tool highly for completeness, relevance, and articulation, achieving a mean CVI of 0.98. This result indicates that the translation and cultural adaptation effectively captured the intended nutritional constructs (25).

The internal consistency reliability of the Arabic sHEI was supported by a Cronbach’s alpha of 0.81, similar to that of other DQ tools with values between 0.67 and 0.89 (24, 38–40). However, lower consistency (Cronbach’s alpha of 0.44) for moderation-related items suggests the need for further refinement of these components. The participation of Saudi nutritionists and dietitians in the translation process contributed to the cultural relevance and clarity of the tool. However, the process also highlighted variability in dietary habits, underscoring the importance of context-specific dietary assessments.

While the original sHEI was designed for young university students in the US (13), it has been successfully used in diverse contexts, such as exploring dietary patterns among adults of African and East Asian backgrounds in the US (41). Although the US dietary guidelines underpin the sHEI, they are aligned with nutrition policies in Saudi Arabia, making this tool applicable within the Saudi context (42). Moreover, the Arabic-translated version of the sHEI can be adapted for use in various Arabic-speaking populations by modifying specific items to reflect national dietary recommendations, dietary patterns, and frequently consumed foods. Such adaptation should follow established cross-cultural adaptation protocols, including linguistic validation and pilot testing to ensure cultural relevance and psychometric validity (43).

A key strength of this study is that it represents the first adaptation and validation of a DQ tool specifically for the Saudi population. The large sample size of young Saudi adults enhances the robustness of the tool’s reliability and applicability for future research and community interventions.

However, this study has certain limitations. First, the sensitivity of the Arabic sHEI to detect dietary changes over time or in intervention-based settings has not yet been tested, which limits its use in longitudinal studies (37). Second, the tool has not been compared to gold-standard dietary assessment methods, such as the original HEI, 24-h dietary recalls, or food frequency questionnaires, which would further establish its validity (13). Third, convenience sampling might have resulted in sampling bias, potentially affecting the representativeness of the results. However, the research team ensured the study instrument was distributed electronically using a snowball sampling without influencing or interfering with participation. Furthermore, the selection of the current sample from university students may limit the generalizability of the findings to the overall young adult population. However, as the original tool was developed and validated in the same age group, our future direction is to validate it among older Saudi and Gulf populations. Therefore, future studies should include more diverse populations from different age groups. Finally, although test–retest reliability was not assessed in the study, an alternative reliability test, such as internal consistency, was conducted, which is commonly used and widely accepted for evaluating the reliability of newly developed instruments (29).

## 5 Conclusion

The Arabic version of the sHEI developed in this study demonstrates strong validity and reliability for assessing overall DQ in young Saudi adults. It is a practical tool that holds significant potential for use in community-based assessments, nutritional interventions, and research settings. However, the Arabic version of the sHEI needs to be used with caution. The authors recommend testing the validity and reliability in other Arab populations and different age groups. Future studies should explore the application of this tool in broader population groups, including both youths and adults, to further establish its utility across diverse contexts.

## Data Availability

The original contributions presented in the study are included in the article/supplementary material, further inquiries can be directed to the corresponding author.
